# Supplementary material for: Side effects of COVID-19 vaccines and perceptions about COVID-19 and its vaccines in Bangladesh: A Cross-sectional study
Source: Vaccine X. 2022 Aug 22;12:100207. doi: 10.1016/j.jvacx.2022.100207 (PMC9394094; doi:10.1016/j.jvacx.2022.100207)
Supplement: Supplementary data 1 [file mmc1.pdf]

Title: Side Effects of COVID-19 Vaccines and Perceptions about COVID-19 and Its Vaccines in Bangladesh

Section A	Eligibility/Basic information		
**Serial No.**	**Question**	**Options**	**Remarks**
**Q1**	**Consent:** Assalamu Alaikum/Adab (Greetings). I would like to request you to take part in a small survey. This study aims to explore the side effects of the COVID-19 vaccines in Bangladesh. The survey includes questions regarding your COVID-19 vaccination and its side effects (if any). It also includes some socio-demographic questions. I hope this information will help the Bangladesh government and policymakers to make a better vaccination strategy to fight off the COVID-19 pandemic. You will not be asked any personal or sensitive questions. You will not be asked your name/identity. The survey may take 4-5 minutes to complete. Please note that your participation in the survey is entirely voluntary. You can reject it anytime during the survey. Do you consent to participate in the study?	1. Yes<br>**2.** No	If Q1=2, then shows “Thank you for your time” and ends the survey.
Q2	Gender	1. Male<br>2. Female	
Q3	(**Eligibility Check**) How old are you (in years)?	1. Less than 12 years<br>2. In between 12 to 17<br>3. In between 18 to 29<br>3. In between 30 to 39	If Q3=1 , then shows “Unfortunately you are not

		4. In between 40 to 49<br>5. In between 50 to 59<br>6. In between 60 to 69<br>7. 70 or above	eligible to take part in this survey as you are under 12, please stop and submit it”
**Q4**	(**Eligibility Check**)<br>Have you taken any Covid-19 vaccine (at least one dose)?	1. Yes<br>**2.** No	
**Q5**	Reason for not taking a vaccine yet	1. Did registration, not yet received<br>2. Did not register, willing to take<br>**3.** I don’t want to take a vaccine<br>**4.** I don’t know<br>**5.** Others (specify)	**If Q4=2**
**Q6**	Why don’t you want a Covid-19 vaccine?	1. It is very difficult to manage a vaccine<br>2. Vaccines aren’t safe and effective<br>3. Vaccines can cause death<br>4. People get infected and die even after taking the vaccines<br>5.Covid-19 isn’t a serious disease, I don’t fear<br>6. Only God (Allah/Vogoban) can save us<br>7. I have no time to go to the Vaccine center<br>8. I don’t know<br>8. Other (specify)	**If Q4=2 &Q5=3**
**Q7**	Why didn’t you register for the Covid vaccine?	1. I don’t know how to register<br>2. I don’t have access to computers/smartphones/internet<br>3. I don’t know what it requires to get a vaccine<br>4. I do not have my NID/BR card or lost the NID/BR<br>5. Other (specify)	**If Q4=2 & Q5=2**
**if Q4=2, show *“Thank you for your time, unfortunately, you are not eligible to take part in this survey, please stop and submit”*, otherwise continue**			
**Section B: Demographics**			

Q8	Your marital status	1. Single<br>2. Married<br>3. Widowed<br>4. Divorced<br>5. Other(specify)	
Q9	Where do you live?	1. Urban area (City corp, municipality)<br>2. Rural area	
Q10	What is your highest completed/running level of education?	1. No formal education (Illiterate)<br>2. Primary completed<br>3. Higher secondary (grade 6-10)<br>4. SSC or equivalent completed<br>5. HSC or equivalent passed<br>6. Undergraduate (Hon’s/MBBS/Degree/Technical or Vocational)<br>7. Graduate (Maters/PhD/Mphil)<br>8. Other (specify)	
Q11	What is your religion?	1. Islam<br>2. Hinduism<br>3. Christianity<br>4. Buddhism<br>5. Other religion (specify)	
Q12	In which of the following categories would you place your monthly household income from all sources before tax and any other deductions? (in Taka)	1. Under 10,000<br>2. 10,000-19,999<br>3. 20,000-29,999<br>4. 30,000–39,999<br>5. 40,000–49,999<br>6. 50,000–74,999<br>7. 75,000 or over<br>8. Don't know	
Q13	What is your occupational status?	1. Service holder (govt/private/RMG)<br>2. Small business (less than 5 employees)<br>3. Large business (5 or more employees)	

		4. Day laborer/Rickshaw/Van/Auto driver<br>5. Motor vehicle driver<br>6. Student<br>7. Housewife<br>8. Unemployed<br>9. Retired/disabled/sick<br>10. Other (specify)	
**Section C**		**Vaccination**	
Q14	Which Covid-19 vaccine did you take?	1. OxfordAstraZeneca (Oxford vaccine)<br>2. Pfizer-BioNTech (Pfizer vaccine)<br>3. Moderna<br>4. Sinopharm<br>5. Sinovac<br>6. Johnson & Johnson<br>7. Sputnik V<br>8. Don’t know the name	If Q4=1, the survey will continue. Otherwise, it will stop after the demographic part.
Q15	Who/which influenced you to take the vaccine?	1. I willingly took it<br>2. My family members/relatives convinced me<br>3. It was compulsory for workplace/educational institution<br>4. Neighbor/village leader inspired me<br>5. Others (specify)<br>6. Don’t know	
**Section D**	**Underlying health conditions of respondents**		
Q.15	Which of the following underlying conditions do you have? (Select all that apply)	1. No underlying conditions<br>2. Diabetes<br>3. Heart disease/Heart attack<br>4. Hypertension/High blood pressure<br>5. Low blood pressure<br>6. Cancer<br>7. Obesity<br>8. Severe allergic problems<br>9. Chronic respiratory diseases (Pneumonia, Asthma, breathing issues)<br>10. Liver/kidney disease	

		11. Anemia<br>12. Other regular health issues (specify)	
Q16	Do you smoke	1. Yes<br>2. No	
Q17	Do you drink (Alcohol)/take illicit substances (Gaja/Yaba, etc.)	1. Yes<br>2. No	
**Section E**	**Side effects (if Q4=1)**		
Q18	Did you have any side effects after taking the vaccine (normal or severe)?	1. Yes<br>2. No	If Q-18=1, then ask all the side-effects related questions
**Now we will ask you some questions about the side effects you faced after taking the covid vaccine**			
Q19	Pain at the vaccination site	1. Severe pain<br>2. Normal pain<br>3. No pain	
Q19.1	How many days did it last (no. of days)		If Q19=1 or 2
Q20	Redness/swelling at the vaccination site (ঈঞ্জেকশনের স্থানে লাল/ফুলা ভাব)	1. Yes<br>2. No	
Q20.1	How many days did it last (no. of days)		If Q20=1
Q21	Fever	1. Normal fever<br>2. Severe fever<br>3. No fever	
Q21.1	How many days did it last (no. of days)		If Q21=1 or 2
Q22	Headache	1. Yes<br>2. No	
Q22.1	How many days did it last (no. of days)		If Q22=1
Q23	Lethargy/tiredness	1. Yes<br>2. No	

Q23.1	How many days did it last (no. of days)		If Q23=1
Q24	Nausea	1. Yes<br>2. No	
Q24.1	How many days did it last (no. of days)		If Q24=1
Q25	Diarrhea	1. Yes<br>2. No	
Q25.1	How many days did it last (no. of days)		If Q25=1
Q26	Cough	1. Yes<br>2. No	
Q26.1	How many days did it last (no. of days)		If Q26=1
Q27	Allergic reaction	1. Yes<br>2. No	
Q27.1	How many days did it last (no. of days)		If Q27=1
Q28	Muscle pain	1. Yes<br>2. No	
Q28.1	How many days did it last (no. of days)		If Q28=1
Q29	Abdominal Pain	1. Yes<br>2. No	
Q29.1	How many days did it last (no. of days)		If Q29=1
Q30	Did you feel anxious after taking the vaccine?	1. Yes<br>2. No	
Q31	Did you experience a decrease in sleep quality?	1. Yes<br>2. No<br>3. Don’t know	
Q32	Did you experience an increase in sleep time?	1. Yes<br>2. No<br>3. Don’t know	
Q33	Did you take any medicine (s) due to the side effects?	1. Yes<br>2. No	
Q34	How serious were the side effects of the vaccine?	1. Not serious, I was able to do all my usual activities	

		2. I couldn't do my usual activities for 1 day<br>3. I couldn't do my usual activities for more than 1 day<br>4. Side effects were enough to get worried<br>5. I needed to get hospitalized/see a doctor<br>6. I don't know	
Q35	How different were the reactions of the two doses?	1. Yet to take the second dose<br>2. Reactions after the first dose were more severe<br>3. Reactions after the second dose were more severe<br>4. There was no difference<br>5. Don't know	
Q36	Has anybody discouraged you from taking the 2nd dose because of the side effect in your first dose?	1. Yes<br>2. No	
**Section F**	**Perceptions about Covid-19 and its vaccines**		
Q37	How safe do you think you are after getting vaccinated?	1. 100% safe, will never get infected with Covid-19<br>2. Feel safe, but still, I could get infected<br>3. No confidence, I can get infected anytime<br>4. Don't know	If Q4=1
Q38	Covid-19 vaccines can protect you from serious COVID-19 illness (hospitalization, oxygen, ventilators, or death)	1. Agree<br>2. Neutral<br>3. Disagree	
Q39	All eligible people should take Covid-19 vaccines	1. Agree<br>2. Neutral<br>3. Disagree	

Q40	Even after getting fully vaccinated, we should maintain safety protocols (wearing mask, washing hands, avoiding gatherings, etc)	1. Agree<br>2. Neutral<br>3. Disagree	
Q41	Govt. and policymakers should make it mandatory for all eligible people to take a Covid-19 vaccine	1. Agree<br>2. Neutral<br>3. Disagree	
Q42	Govt. and specialists should decide which Covid vaccine is suitable for whom (considering age, gender, health conditions, etc)	1. Agree<br>2. Neutral<br>3. Disagree	
Q43	People should have a preference in choosing which Covid vaccine to take	1. Agree<br>2. Neutral<br>3. Disagree	
**N1**	If available, how likely are you to allow children of your family (5 or older) to take Covid vaccines?	Not at all likely<br>Somewhat likely<br>Extremely likely	
N4	If available, how likely are you to allow older people of your family (70 or older) to take Covid vaccines?	Not at all likely<br>Somewhat likely<br>Extremely likely	
N6	How likely are you to wear a mask when you are outside/in public transport/shops/public places?	Not at all likely<br>Somewhat likely<br>Extremely likely	
N7	How likely are you to recommend getting the COVID-19 vaccine to others?	Not at all likely<br>Somewhat likely<br>Extremely likely	
N8	How concerned are you about getting COVID-19?	Not at all concerned<br>A little concerned<br>Moderately concerned<br>Very concerned	
N9	How do you feel about the amount of information	I'm not getting enough information.	

	on COVID-19 and its vaccines that you are getting?	I'm getting<br>enough information.<br>I'm getting too<br>much information.	
N10	What are your sources of information about Covid-19 and its vaccines (select all that apply)?	Social media (facebook/instagram/twitter, etc)<br>TV/Radio/Newspaper<br>Family/Friends<br>Neighbors/Other people<br>Local health officials/Covid websites<br>WHO/CDC/FDA<br>Others (specify)	
N11	How difficult it is for general people to get Covid vaccines in Bangladesh?	Very easy<br>Somewhat easy<br>Somewhat difficult<br>Very difficult<br>Not sure	
N12	How likely is it that Covid-19 spreads all over Bangladesh again?	Not at all likely<br>Somewhat likely<br>Extremely likely	
Q44	Do you have any idea about the cost of vaccines that the government is spending	1.Yes<br>2.No	
Q45	How much cost for each dose		If Q 44 is 1
